# Improved Draft Genome Sequence of a Monoteliosporic Culture of the Karnal Bunt (Tilletia indica) Pathogen of Wheat

**DOI:** 10.1128/genomeA.00015-18

**Published:** 2018-05-17

**Authors:** Anil Kumar, Pallavi Mishra, Ranjeet Maurya, A. K. Mishra, Vijai K. Gupta, Pramod W. Ramteke, Soma S. Marla

**Affiliations:** a Department of Molecular Biology and Genetic Engineering, College of Basic Sciences and Humanities, G. B. Pant University of Agriculture and Technology, Pantnagar, India; b ICAR-Agricultural Knowledge Management Unit, Indian Agricultural Research Institute, Pusa Campus, New Delhi, India; c Department of Computational Biology and Bioinformatics, Sam Higginbottom University of Agriculture, Technology and Sciences, Allahabad, India; d Department of Chemistry and Biotechnology, ERA Chair of Green Chemistry, Tallinn University of Technology, Tallinn, Estonia; e Department of Biological Sciences, Sam Higginbottom University of Agriculture, Technology and Sciences, Allahabad, India; f Division of Genomic Resources, ICAR-National Bureau of Plant Genetic Resources, New Delhi, India

## Abstract

Karnal bunt of wheat is an internationally quarantined fungal pathogen disease caused by Tilletia indica and affects the international commercial seed trade of wheat. We announce here the first improved draft genome assembly of a monoteliosporic culture of the Tilletia indica fungus, consisting of 787 scaffolds with an approximate total genome size of 31.83 Mbp, which is more accurate and near to complete than the previous version.

## GENOME ANNOUNCEMENT

Karnal bunt (KB) of wheat crops was first discovered in Karnal, India, in 1931 (1) and is caused by the smut fungus Tilletia indica, a basidiomycete belonging to the subdivision Ustilaginomycotina. KB has become a major disease which hampers international wheat trade due to quarantine regulations imposed by several countries (2). In order to understand the offensive and defensive mechanism(s) in the wheat-T. indica interaction at the molecular level, complete genomic information of fungus is of paramount importance. To date, attempts have been made by several research groups to decipher the genome sequence using next-generation sequencing platforms (3–5). The redundancy in the genetic profiling of the fungus T. indica necessitates the refinement of available genome sequences of monoteliosporic cultures of T. indica for better appreciation of fungal biology and disease management (6).

The Karnal bunt fungus T. indica assemblies for isolates DAOM 236416 (4) and RAKB_UP_1 (5) were retrieved from the NCBI database (https://www.ncbi.nlm.nih.gov) and were preprocessed accordingly for the reconciliation algorithms.

The Illumina and PacBio sequence reads of the TiK (Tilletia indica Karnal) isolate (3) were quality checked using FastQC version 0.11.5 (7) and fastQValidator version 0.1.1 (8), and the reads were repaired using BBmap version 37.66 (9). Adapter sequences along with low-quality bases were removed using PRINSEQ version 0.20.4 (10). The high-quality Illumina and PacBio reads were *de novo* assembled using hybridSPAdes version 3.11.0 (11). The improved draft version of the assembly was generated by using Metassembler version 1.5 (12) by merging the draft monoteliosporic sequence-based assemblies from the DAOM 236416 and RAKB_UP_1 isolates with the improved and reassembled hybrid assembly. Genome assembly gap filling and polishing on the merged assembly were done by GapFiller version 1.10 (13, 14) and Pilon version 1.22 (15), respectively. The improved merged draft assembly consists of 787 scaffolds with a total genome size of 31,836,179 bp (*N*_50_, 80,772 bp), with a GC content of 54.79% and an average coverage depth of at least 107×, which covers higher genome statistics (high *N*_50_ value, maximum contig length, and number of minimum contigs in scaffolding with respect to the high depth of coverage, which leads to an improved genome assembly) than the other published draft genome sequences of KB pathogen.

For comprehensive genome annotation, the improved draft scaffolds were first repeat masked using RepeatMasker version 4.0.7 (16), followed by genomic annotation using MAKER version 2.31.9 (17), AUGUSTUS (18–20), and SNAP (21). A total of 9,209 protein-coding gene models and 0.21% repeat elements were observed for the improved TiK isolate. The improved draft version was also screened for the presence of simple sequence repeat (SSR) loci using MISA (22), and 5,734 SSR loci were identified. The most abundant SSR type in the genome was trinucleotides, with 2,449 (42.71% of all SSRs). This is the first improved genome sequence of a monoteliosporic culture of the KB pathogen of wheat from the order Georgefisheriales (Exobasidiomycetes).

The availability of a near-complete, more accurate, and nonredundant genome sequence serves as baseline data to provide ample opportunities to understand the pathogenic mechanisms as the model for the identification of the fungal pathogenic determinants involved in disease development, which will be used for devising effective crop protection strategies as part of the development of resistant wheat cultivars showing immunity against KB (23).

### Accession number(s).

This improved whole-genome shotgun project has been deposited at DDBJ/ENA/GenBank under the accession number PKQB00000000. The version described in this report is version PKQB01000000 for TiK.
